# Regionally high risk increase for precipitation extreme events under global warming

**DOI:** 10.1038/s41598-023-32372-3

**Published:** 2023-04-05

**Authors:** Cristian Martinez-Villalobos, J. David Neelin

**Affiliations:** 1 Faculty of Engineering and Science, Universidad Adolfo Ibáñez, Santiago, Chile; 2 Data Observatory Foundation, Santiago, Chile; 3 Department of Atmospheric and Oceanic Sciences, University of California, Los Angeles, Los Angeles, CA USA

**Keywords:** Atmospheric science, Climate change, Hydrology, Climate sciences, Environmental sciences, Hydrology

## Abstract

Daily precipitation extremes are projected to intensify with increasing moisture under global warming following the Clausius-Clapeyron (CC) relationship at about $7\% /^\circ {\text{C}}$. However, this increase is not spatially homogeneous. Projections in individual models exhibit regions with substantially larger increases than expected from the CC scaling. Here, we leverage theory and observations of the form of the precipitation probability distribution to substantially improve intermodel agreement in the medium to high precipitation intensity regime, and to interpret projected changes in frequency in the Coupled Model Intercomparison Project Phase 6. Besides particular regions where models consistently display super-CC behavior, we find substantial occurrence of super-CC behavior within a given latitude band when the multi-model average does not require that the models agree point-wise on location within that band. About 13% of the globe and almost 25% of the tropics (30% for tropical land) display increases exceeding 2CC. Over 40% of tropical land points exceed 1.5CC. Risk-ratio analysis shows that even small increases above CC scaling can have disproportionately large effects in the frequency of the most extreme events. Risk due to regional enhancement of precipitation scale increase by dynamical effects must thus be included in vulnerability assessment even if locations are imprecise.

## Introduction

Events of extreme precipitation are among the costliest natural disasters^1,2^. They are associated with flooding^3^, damage to infrastructure^4^ and cost in lives. In the United States alone, extreme precipitation events have caused more than 200 billion damages during 1988–2017, with an increasing trend in costs^2^ as these events become more frequent^5–7^. As a baseline, previous studies commonly assume that the intensity of extreme precipitation increases with warming following the Clausius-Clapeyron (CC) relationship at about $7\%$ for each additional $^\circ {\text{C}}$ of warming^8–11^. However, it has been progressively recognized that changes in moisture alone do not explain the expected future pattern of precipitation extremes changes, and that changes in wind circulation also play an important role^11–16^. This implies that at a given location, increases in the intensity and frequency of extreme precipitation may deviate from the CC scaling expectations. In this article we use theory that connects local precipitation probability distributions with the underlying moisture budget^11,14,16,17^ to better understand the disproportionate impacts that even small deviations from the CC scaling may have in future increases in daily precipitation extremes intensity and frequency.

Although commonly used, the CC scaling is loosely defined —there is no extreme index specification attached to its use. Past studies have reported different scalings for different extreme indices^10,18–22^, with more extreme percentiles or longer return periods events generally increasing faster in intensity^18,19,23,24^ and also frequency^7,14,25–27^. For example, Ref.^23^ projects an ensemble mean increase of $6.5\% /^\circ {\text{C}}$ for the 99.5th percentile, and $9.2\% /^\circ {\text{C}}$ for the 99.9th percentile of daily precipitation in Australia. This lack of apparent convergence, either to a CC or a super-CC scaling, of projected changes of different extreme indices leads us to ask whether there is a parsimonious explanation for this behavior.

At the same time, global warming projections of increases in the intensity and frequency of the largest events are also subject to the most uncertainty^21^. An uncertain future also means uncertainty in decision making for, e.g., how we design infrastructure that can withstand increasingly larger and frequent storms^4,28^, and how we rethink our cities in general^29^. Given the high impact of these events, an additional motivation of this study is to the search for ways to reduce this uncertainty.

Here, we leverage theory for precipitation probability distributions to improve estimates of changes of precipitation extremes and to identify the occurrence of large changes at the regional scale as follows. First, we introduce the different regimes of daily precipitation probabilities in observations and in models of the Coupled Model Intercomparison Project (CMIP6) ensemble. We show that changes in a key precipitation scale that controls one of the regimes underlies changes in both intensity and frequency of precipitation extremes. Then, we apply knowledge of these regimes to reduce the projected uncertainty in an important measure of daily precipitation frequency changes: risk ratios. We then show that regional occurrences of super-CC scaling are widespread in the tropics. Finally, we explore the large consequences that even small increases above super-CC scaling have on the projected frequency of the most extreme daily precipitation events.

## Results

### Two regimes of daily precipitation probability

**Figure 1:**
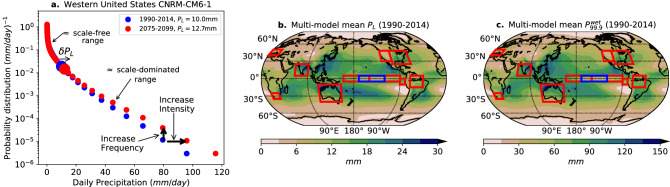
(**a**) Example of simulated daily precipitation probability distributions in the historical and global warming (SSP5 8.5) runs in the CNRM-CM6-1 model for the Western United States (30$^\circ$N–48$^\circ$N, 103$^\circ$W–124$^\circ$W). The plot showcases the two leading order probability distribution regimes: an approximately scale-free range controlling the probability of low and moderate daily precipitation values, and a scale-dominated range controlling the large-event tail. The scale $P_{L}$ is a key parameter controlling the intensity and frequency of extreme daily precipitation events. (**b**) Multi-model mean of $P_{L}$ in the CMIP6 historical run (1990–2014). (**c**) Multi-model mean of the 99.9th wet-day daily precipitation percentile in the CMIP6 historical run (1990–2014). Red boxes show the location of regions used to exemplify behavior in this and remaining figures. The Niño 3.4 region is shown in blue as it overlaps Niño 3 and Niño 4 regions. Maps were generated using Python’s^30^ Matplotlib^31^ Basemap^32^ Toolkit.

The probability of daily precipitation over wet days follows two physically distinct regimes^16^, as seen in observations^7,33,34^, and models^11,27,35–37^. For low and moderate daily precipitation *P* values the probability density decreases following an approximately scale-free range up to a characteristic precipitation scale $P_{L}$. For precipitation larger than $P_{L}$, the probability decreases follow a different behavior dominated by this scale (Fig. 1a). Thus, $P_{L}$ exerts a leading control on the probability of extreme wet-day percentiles^34,35^. This is illustrated as a function of space comparing the precipitation scale $P_L$ (Fig. 1b) to the local 99.9th percentile of daily precipitation (Fig. 1c).

A family of distributions featuring these two regimes may be written $f\propto P^{-\tau _{P}}F(-P/P_{L})$. Here, the scale free range is represented by a power law $P^{-\tau _{P}}$ (Fig. 1a), where $\tau _{P}$ is an exponent usually between 0 and 1^16^, and the scale-dominated range is represented by the general dependence $F(P/P_{L})$. A simple and useful case of this is the Gamma distribution with $F(P/P_{L})=exp(-P/P_{L})$^16,38,39^ with the scale-dominated range represented by an exponential tail with characteristic rate of decrease $P_L$. We use properties of this distribution to illustrate a number of points, although the key results depend simply on existence of the scale-dominated regime, as elaborated in Methods. Caveats on the Gamma distribution per se and discussion of the relation of the precipitation distribution examined here to extreme-value approaches are also provided in Methods.

As in previous studies^33,34,37^, here we estimate $\tau _{P}$ and $P_{L}$ using the first two moments of the distributions (see Methods). Both $\tau _{P}$ and $P_{L}$ have physical connections to the underlying moist dynamics. Specifically, $\tau _{P}$ depends critically on behavior on dry times, with regions with few precipitating events per wet day showcasing steeper power law ranges (larger $\tau _{P}$)^16,37^. The precipitation scale $P_{L}$, on the other hand, is set by dynamics occurring in wet periods and scales with the amplitude of moisture convergence fluctuations during raining times^11,16^. Thus, $P_{L}$ and consequently wet-day extreme percentiles do not simply depend on moisture levels but also on convergence variance during wet times. It has been further argued that at a given percentile of precipitation an increase in moisture requires a change in convergence to simultaneously satisfy both moisture and thermodynamic equations^11^ except under particular circumstances. This implies that deviations from a CC scaling are not only possible but expected. However, if there is intermodel uncertainty in the convergence feedback yielding super-CC scaling, it may be underestimated in traditional multimodel ensemble averaging.

**Figure 2:**
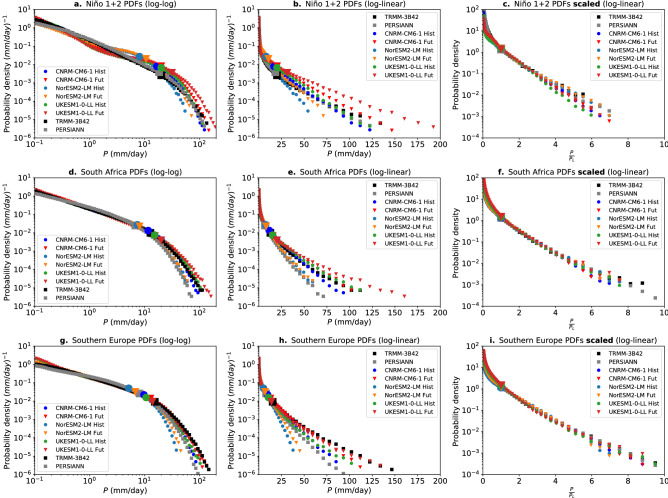
(**a**) Daily precipitation probability distributions in 3 models (color; CNRM-CM6-1, NorESM2-LM, UKESM1-0-LL) under historical (1990–2014) and SSP5-8.5 (2075-2099) forcing conditions in the Niño 1+2 area (10$^\circ$S-0$^\circ$S, 90$^\circ$W-80$^\circ$W). Black and gray curves show observational estimates from TRMM-3B42^40^ (1998-2018) and PERSIANN^41^ (1983–2017) datasets respectively. Both axes are logarithmic. (**b**) Same as (**a**) but with a linear x-axis. (**c**). Same as (**b**) but using a scaled coordinate $\frac{P}{P_{L}}$. Details on the rescaling methodology are provided in Ref.^35^. Note that for illustration purposes, here both historical and SSP5-8.5 precipitation are rescaled by their own precipitation scales $P_{L}$, but to calculate the improved estimation of risk ratios (Figs. 3 and 6 ) both historical and global warming precipitation data are rescaled by the historical precipitation scale. (**d**) Same as (**a**) but over South Africa (25S–35S, 15E–30E). (**e**) Same as (**b**) but over South Africa. (**f**) Same as (**c**) but over South Africa. (**g**) Same as (**a**) but over Southern Europe (40N–50N, 0E–20E). (**h**) Same as (**b**) but over Southern Europe. (**i**) Same as (**c**) but over Southern Europe.

**Figure 3:**
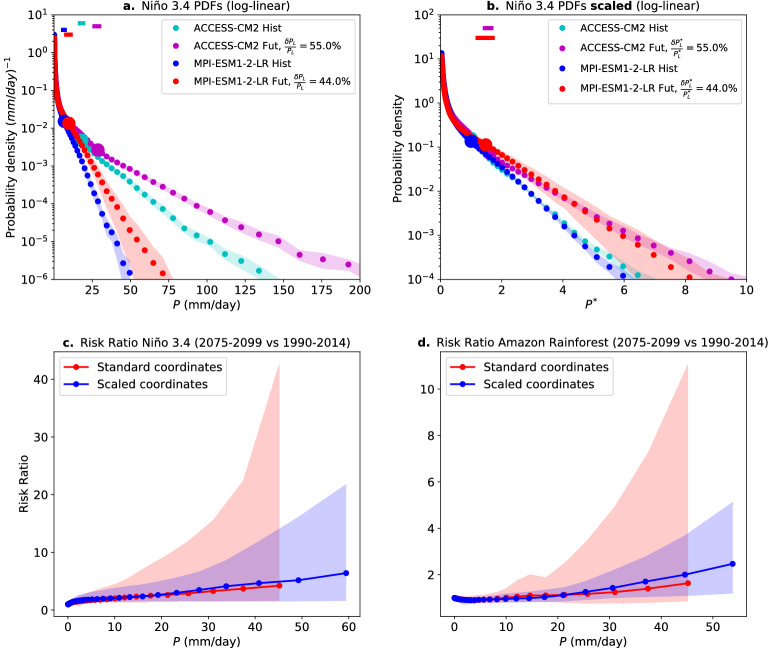
(**a**) To illustrate effects of the rescaling procedure, historical and global warming daily precipitation probability distributions are shown for two models that bracket the range of simulated historical $P_{L}$ in the Niño 3.4 region. Shading denotes the 5–95th percentile range of each model’s PDF from an ensemble of bootstrap realizations (see Methods). The 5th-95th percentile of $P_{L}$ estimations are shown in the top left (corresponding $P_L$ values are shown on the respective PDFs as large dots; note these dots are larger than the error bars). (**b**) Same probability distributions as in (**a**) but in scaled coordinates (i.e., $\frac{P}{P_{L}^{his}}$). This allows an approximate collapse of the tails of the historical distributions, reducing the source of uncertainty due to different model simulations of historical $P_{L}$. Note that the global warming distributions need not collapse, as their large event tail slope (in linear-log coordinates) is given by the simulated increase in $P_{L}$ (noted in legend). These differences in $\frac{\delta P_{L}}{P_{L}}$ contribute to the intermodel spread in risk ratios shown in blue in the panels below. (**c**) Risk ratios (2075–2099 vs. 1990–2014) calculated in standard coordinates (red) and scaled coordinates (blue) in the Niño 3.4 region (5$^\circ$S-5$^\circ$N,120$^\circ$W-170$^\circ$W). Solid lines represent the multi-model mean and the shading encompasses the 5th-95th percentiles across models. (**d**) Similar to (**a**), but for the Amazon Rainforest region (15$^\circ$S-5$^\circ$N,45$^\circ$W-70$^\circ$W). Only bins where the 5th percentile (across models) contains at least 50 counts are displayed.

### Reducing the inter-model spread of projected changes in extreme precipitation frequency

In addition to often displaying the largest projected increases, projections of changes in precipitation extremes are also subject to the largest degree of uncertainty^21,42^. In the case of changes in frequency, there are three main sources that explain the inter-model spread of risk ratios in the large event range: **a.** the inter-model spread of the precipitation scale $P_{L}$ in historical simulations^36,37^, **b.** the inter-model spread in the simulation of $\delta P_{L}$, and **c.** model deviations of the daily precipitation probability distribution from the observed form^36,37,43^. To reduce the uncertainty due to source **a**, we introduce a scaled coordinate $P^{*}=\frac{P}{P_{L}}$^35^. Figure 2 illustrates the effects of rescaling the precipitation distribution by its dominant scale for three selected regions. The left two columns contain the same information but presented in a manner that highlights the two behavior ranges. Figure 2a,d,g presents the PDFs on a log-log scale to highlight the approximately scale-free range for low to medium intensity. In some regions, e.g. South Africa, the models reproduce the observed scale-free (approximately power-law) behavior; in others, e.g. Niño1+2, the models exhibit noticeable departures from the observed form in this range. On the log-log plot, the transition to the second behavior regime is seen as a steeper drop in probability beginning near the precipitation scale $P_L$ (which is thus alternately referred to as the cutoff scale). Figure 2b,e,h presents the PDFs on a log-linear scale to highlight the behavior of the medium to high-intensity range. The leading differences among the models arise from differences in the slope of the PDFs in this range, i.e., from differences in characteristic precipitation scale $P_L$. The right-hand column (Fig. 2c,f,i) shows the results of rescaling by an estimate of $P_L$. In agreement with ref.^35^, it yields a good collapse of the medium to high intensity range of the distribution through 2-3 orders of magnitude of the PDF. The very most extreme events still exhibit some uncertainty due to a combination of imperfections in the moment estimator of $P_L$ (seen as departures of the slope from -1; estimation error is elaborated in Methods) and slight differences in shape among the models. The slight curvature seen at very high $P/P_L$ in Fig. 2c,f,i for some cases indicates departures of $F(P/P_L)$ from exponential. Nonetheless, the much closer similarity in the medium-high range in the right hand column compared to the center column illustrates: 1) the extent to which certain aspects of the uncertainty can be reduced using information about the historical $P_L$ for each model and region; and 2) the extent to which changes in the future PDF relative to the historical can be accounted for just by the information contained in the relative values of $P_L$.

We now apply this scaling to reduce the uncertainty in an important measure of changes in frequency of extremes, risk ratios^7,14,34,44–46^. A risk ratio $r(P_{r})$ is defined as the ratio of the probability (conditioned on wet-day occurrence) of daily precipitation larger than $P_{r}$ in a global warming run compared with the historical case. As an example, a risk ratio of 3 for $P_{r}=100$mm implies that wet-days with $P>100$mm are three times more frequent in the future compared with historical conditions. When the range of risk ratios is naïvely calculated from the PDF of each model as a function of precipitation, differences in the historic distribution contributes significantly to the spread. Calculating risk ratios in each model’s scaled coordinate $P^{*}$ using the historical $P_L$ substantially removes this source of uncertainty while preserving the fractional changes in $P_{L}$ (which are the same in standard and scaled coordinates).

To illustrate how this is accomplished, the top two panels of Fig. 3 show two individual models’ PDFs in standard (Fig. 3a) and scaled coordinates (Fig. 3b) in the Niño 3.4 region. The difference between the models’ historical $P_L$ (here MPI-ESM1-2-LR; $P_{L}^{his}=5.7$mm/day, and ACCESS-CM2; $P_{L}^{his}=16.8$mm/day) can be larger than the changes seen for each model under global warming, and substantially larger than the uncertainty range in estimation of the PDF and of $P_L$ for each model. The scaling effectively removes this difference in the historical (Fig. 3b), allowing the relative increase in the PDFs under warning to be more closely compared. Effectively, the scaling improves the intermodel matching of the intensity range where risk ratios start to considerably increase, above each model’s historical precipitation scale (see eq. 3 below as well), which in scaled coordinates is equal to 1 in all models. In some cases, the improvements yielded by this matching are modest (e.g., Fig. S1 showing Southern Europe, India and Australia). This occurs partly because the models are already well aligned along the intensity axis (i.e., the spread in the historical simulation of $P_{L}$ is small), but in other cases improvements can be substantial. Furthermore, intermodel comparisons without scaling tend to be limited by the models with the smallest historical $P_L$ (since these are the first to reach very low counts with increasing intensity). By placing the models on a more equal footing, the scaled coordinate tends to remove this effect, allowing the multi-model analysis to extend to historically less-frequent events with larger intensities. "Consequences of super-CC scaling for risk ratio" section Consequences of super-CC scaling for risk ratio’ expands on this with analytic results.

Figure 3c,d shows the range of projected risk ratios in the Niño 3.4 and Amazon Rainforest regions using standard and scaled coordinates. For this comparison, risk ratios in scaled precipitation coordinates are rescaled by the multi-model mean historical $P_{L}$. To correct the ensemble bias, one could also rescale by an observed historical value of $P_{L}$. Here, the solid lines represent the multi-model mean in each case, with the shading denoting the 5th-95th percentile across models. We observe a reduction in the uncertainty range using scaled coordinates in both cases, especially in the large event range. In the Amazon Rainforest, for example, the projected 5th-95th percentile of changes in the frequency of precipitation above 45mm ranges from 0.9 to 11.1 in the standard calculation. Using a scaled coordinate, on the other hand, reduces this range of frequency increases to a factor of 1.1–3.7 (Fig. 3d). The reduction is even larger in the Niño 3.4 region, with a range of 1.5–42 times increases in frequency above 45mm in the standard case, compared with a range of 1.5 to 12 times increases in frequency using scaled coordinates (Fig. 3c). A significant benefit is the increased ability to do multi-model estimates for low probability events because these are much better aligned across the model ensemble. In Fig. 3, this is seen in the extended range of precipitation values for which we can make projections.

### Understanding changes of daily precipitation intensity under global warming

Previous research has shown different responses of low and medium percentiles of precipitation under global warming depending on the region^9,10^. For extreme precipitation there is a tendency for the most extreme percentiles or longest return periods to increase the most in model projections under global warming^10,19–21^. We investigate this behavior by assessing how global warming changes in the power law range exponent $\tau _{P}$ and precipitation scale $P_{L}$ (Fig. 1a) affect changes in percentiles across the whole intensity range. In most regions the power law range gets steeper (Fig. S2a), consistent with fewer precipitation accumulation events (from precipitation onset to termination) during wet days^16^, and with a stronger extreme precipitation response under global warming compared to the mean^9,47^ (Fig S2b; see methods).

To the extent that global climate models can correctly simulate the shape of observed daily precipitation probability distributions, a wet-day percentile $P_{q}^{wet}$ can approximately be expressed as

1 $$\begin{aligned} P_{q}^{wet}=P_{L}\Gamma ^{-1}\left( 1-\tau _{P},1-\frac{q}{100}\right) , \end{aligned}$$

where $P_{L}=\frac{\sigma _{P}^{2}}{{\bar{P}}_{wet}}$, $\tau _{P}=1-\frac{{\bar{P}}_{wet}}{P_{L}}$, ${\bar{P}}_{wet}$ is the mean precipitation over wet days, $\sigma _{P}^{2}$ is the daily precipitation variance over wet days, $\Gamma (y,z)=\frac{1}{\Gamma (y,0)}\int _{z}^{\infty }x^{y-1}exp(-x)dx$ is the incomplete Gamma function, and $\Gamma ^{-1}(y,z)$ is the inverse of the incomplete Gamma function. A future fractional change of a wet-day percentile, thus, can parsimoniously be understood by changes in the power law range and precipitation scale given by

2 $$\begin{aligned} \frac{\delta P_{q}^{wet}}{P_{q}^{wet}}=\gamma (\delta P_{L})+G(\delta \tau _{P}), \end{aligned}$$

where *G* is a function (explicitly given in Eq. 9 in "Methods" section) that depends on changes in the power law range and which dominates the response of low and moderate percentiles, and $\gamma (\delta P_{L})=\frac{\delta P_{L}}{P_{L}}$ is the fractional change in the precipitation scale which dominates the changes in extreme percentiles^33,35^. The function *G* is such that if the power law range gets steeper under global warming, then extreme percentiles increase faster than low percentiles. In our results we show a modified version of the previous equation (see methods) of similar form (see eq. 10), which takes into account the “left-censoring” of the distribution needed to avoid the “drizzle problem”^37,48–50^ (see Methods). A similar equation to (2) can be written for changes in all-day percentiles (equation 12), with the added complication that changes in the fraction of wet days (Fig. S2c) also enter^51^.

**Figure 4:**
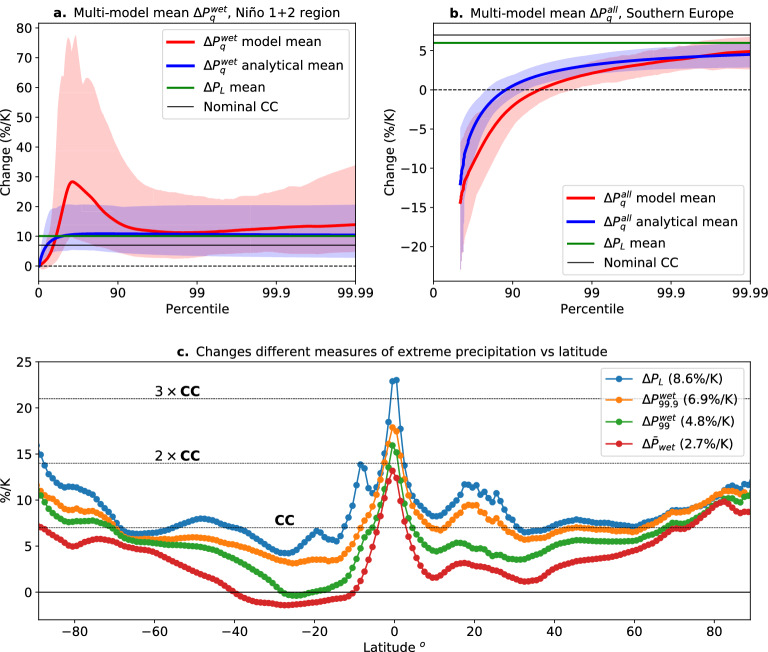
(**a**) Change in left-censored wet-day percentiles between 2075-2099 compared with 1990-2014 in the Niño 1+2 region (10$^\circ$S-0$^\circ$S, 90$^\circ$W-80$^\circ$W) where some models depart from observed PDF shape. Solid red represents the multi-model mean and shaded-red the 5–95th percentile across models. Solid blue represents the multi-model mean and shaded-blue the 5–95th percentile across models of the analytical change (see Methods) in left-censored wet-day percentiles. Changes in these percentiles are given by each model change in $P_{L}$ (multi-model mean shown as a horizontal green line) and $\tau _{P}$ (see Eq. 2 and methods). The nominal CC change is shown as a horizontal black line. (**b**) Same as (**a**) but for changes in left-censored all-day percentiles in the Southern Europe region (40$^\circ$N-50$^\circ$N, 0$^\circ$E-20$^\circ$E). (**c**) Multi-model mean of the zonal average fractional change in $P_{L}$, $P_{99.9}^{wet}$, $P_{99}^{wet}$, and ${\bar{P}}_{wet}$ between 2075–2099 and 1990–2014. Numbers in parentheses show the multi-model mean global change in these quantities. Horizontal lines representing nominal *CC*, $2\times CC$, and $3\times CC$ scaling are shown for reference. In all cases change is normalized by the average increase in global temperature in each model prior to aggregation.

**Figure 5:**
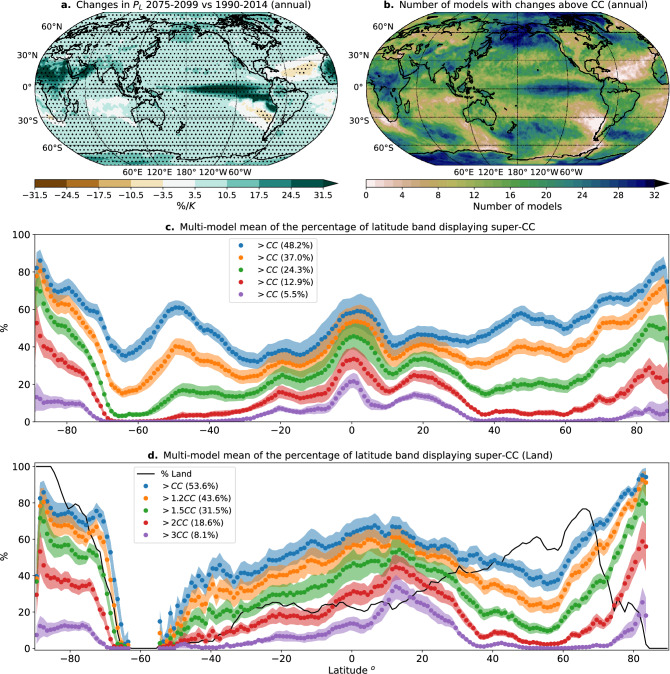
(**a**) Multi-model mean fractional change of the precipitation scale comparing 2075–2099 vs. 1990–2014. Change is normalized by each model global mean temperature change prior to aggregating. Dots show regions in which at least 80% of the models agree in the sign of the change. (**b**) Number of models (out of 32) that display increases in $P_{L}$ (2075-2099 vs 1990-2014) above the nominal CC scaling of $7\%/$K. (**c**) Multi-model mean of the fraction of latitude band displaying increases above *CC* ($7\%/$K), $1.2\times CC$, $1.5\times CC$, $2\times CC$ and $3\times CC$ as a function of latitude. Numbers in parentheses are global averages. (**d**) Same as (**c**) but only considering land points. The solid black line represents the percentage of land in the latitude band. Shading in (**c**,**d**) denotes the 5th-95th percentile of the estimation of the mean from a bootstrap ensemble (see Methods). Maps were generated using Python’s^30^ Matplotlib^31^ Basemap^32^ Toolkit.

We can use these curves to interpret the behavior of the models as function of percentile. Furthermore, the models tend to have errors with respect to observations in the low to intermediate intensity regime^37^ whereas the theoretical curves are substantially better matched to observations. Thus, where the models depart from the theoretical curve it may be taken as an indication of aspects for which model behavior is suspect. Figure 4a,b show examples of CMIP6 (red) and theoretical (blue) changes in wet-day (Fig. 4a) and all-day (Fig. 4b) percentiles across the whole intensity range in two regions with contrasting changes in power law ranges. In the Niño 1+2 region (Fig. 4a) the power law range stays relatively constant with warming (a $\tau _{P}$ ensemble mean estimate of 0.73 for historical and 0.70 for future conditions), while in Southern Europe (Fig. 4b) the power law range gets steeper with warming (a $\tau _{P}$ ensemble mean estimate of 0.52 for historical and 0.58 for future conditions), consistent with fewer projected precipitating events. This translates to a similar increase across the intensity range (for low and high percentiles) in the Niño 1+2 region (Fig. 4a) and smaller increases for low percentiles compared to high percentiles in Southern Europe (Fig. 4b). We note that the probability bump in CMIP6 models at low-medium percentiles in the Niño 1+2 region (Fig. 4a, red curve) occurs due to many models simulating more complex probability distributions than observed (e.g., Fig. 2a), with likely spurious peaks in the scale-free range for that geographic region that are not present in observations^37^. In Southern Europe, a case representative of most regions, percentiles increase the most the more extreme they are. This occurs for both the multi-model mean and the theoretical estimation, although in a smoother way in the latter case. For a steepening of the power law range (2) predicts that fractional increases in $P_{L}$ ($\gamma$, which may well surpass a CC scaling, see Fig. 5a) act as an upper limit for increases in the most extreme percentiles (see Methods). Noting that the power law range is expected to steepen in most regions (Fig. S2a), our theoretical intuition is corroborated by the zonally averaged changes of different measures of extreme precipitation (${\bar{P}}_{wet}$, $P_{99}^{wet}$, $P_{99.9}^{wet}$ and $P_{L}$) in Fig. 4c. Here we can see that, independent of latitude, the more extreme the percentile the larger the increase, and that changes in $P_{L}$ provide an estimator for an upper limit for changes of the most extreme percentiles.

### Changes in precipitation scale and super-CC regions

Previous work^11,14,16^ has used a hierarchy of stochastic models based on the moisture budget to identify the physical processes that govern the behavior of the precipitation scale parameter $$P_{L}$$. They have identified the size of moisture convergence fluctuations within precipitation events as the key variable that determines the value of $$P_{L}$$, and consequently the intensity of precipitation extremes. Under global warming, increases in temperature yields increases in moisture, which in absence of changes in circulation, naturally leads to increases in $$P_{L}$$. These increases can be reinforced or offset by local changes in convergence, which are seen as changes in $$P_L $$ that differ from CC scaling.

Projected CMIP6 global warming changes in the precipitation scale $P_{L}$ are mostly positive, in agreement with theoretical expectations. An increase in $P_{L}$ stretches the large event tail, implying increases in both intensity and frequency of extreme events (Fig. 1a). This implies that the “biggest-get-bigger” effect^14^, that has been shown to occur for precipitation event sizes^11,14^ also apply for daily precipitation extremes. Since $P_{L}$ is a predictor of extreme percentiles^35^, the regional pattern of changes of this scale (Fig. 5a) has counterparts with previous percentile-based^13,15^ or annual maximum-based^52,53^ work. For the conventional multi-model ensemble mean most of the regions have increases ranging between $3.5\%/K$ and $10.5\%/K$, with exceptions mainly in the central and eastern Tropical Pacific (consistent with Ref.^15,54–56^) and the Sahel and Southern Sahara that display super CC behavior (in agreement with Ref.^57^). There are expected decreases in some subtropical regions due to enhanced divergence, including the coast off North and Central Chile and North and South subtropical Atlantic.

Another traditional measure, here adapted to $P_L$ for prevalence of projected super CC behavior in CMIP6, is shown in Fig. 5b: a map of the number of models (out of 32) projecting end of the century increases above nominal CC. Focusing first on regions of high agreement, this measure gives similar information as Fig. 5a for regions like the Central Tropical Pacific and the Sahel, but differs in some others. For example, agreement among models in super CC changes in the North Pacific (including Alaska), as well as in storm-track regions in the Southern Hemisphere are apparent. Changes in the region traditionally associated with El Niño phenomenon (central and eastern tropical Pacific) are about four times as large as it might be expected simply by increases in moisture, consistent with increases in moisture convergence associated with a Niño-like future^58,59^, although other oceanic processes may also be important^55,56,60^. The agreement in this region is robust for the central Pacific, and less so for the eastern tropical Pacific, where fewer models agree on the magnitude of the changes. This suggests less certainty in the future of Coastal El Niño^61,62^ and canonical eastern Pacific ENSO events (cf.^54^). Turning to regions of moderate agreement, Fig. 5b contains widespread regions where over 2/3 of the models exhibit super-CC behavior. A different measure is necessary to distinguish whether there might be substantial super-CC behavior missed by these point-by point average or agreement measures.

Individual models exhibit many super CC regions, but they may not be the same from model to model. Thus, in a traditional ensemble mean they may be averaged out. To test the hypothesis that models are commonly exhibiting regional super-CC behavior without necessarily agreeing on the location, Fig. 5c shows the fraction of each latitude band for which increases exceed specified multiples of nominal CC—1.2CC, 1.5CC, 2CC, and 3CC—averaged over the multi-model ensemble. Corresponding fractions for the global average of grid points exceeding each value (area weighted, computed for each model and then averaged over the ensemble) is given in the legend. Fig. 5d gives the corresponding behavior computed only over land points. The super-CC behavior above the nominal $7\%/$K has a somewhat expected latitudinal dependence, with peaks in the deep tropics and storm-tracks regions in both hemispheres, and a decrease in the subtropics (Fig. 5c). Apparent super-CC behavior at high-latitudes is likely due in part to polar amplification of temperature change relative to the global average^63^. Conversely, low values over the southern ocean likely reflect reduced warming. Defining CC relative to regional-average temperature increase could be considered, but for purposes here we are interested in increases in precipitation probability beyond that of a simple scaling based on global average temperature, for which nominal-CC is more useful. Globally, 7%/K is not far from the median increase. The fraction of points exceeding larger multiples of 7%/K drops for higher multiples but the rate of decrease differs between mid-latitudes and tropics. Over mid-latitudes there are almost no points exceeding 3CC (21%/K) and only about 5% exceed 2CC. Over the tropics, however, a substantial range of latitudes has over 15% of points exceeding 3CC, and 25% exceeding 2CC, with a larger fraction over land for both thresholds. Roughly 40% of tropical land points exceed 1.5CC and half exceed 1.2CC.

**Figure 6:**
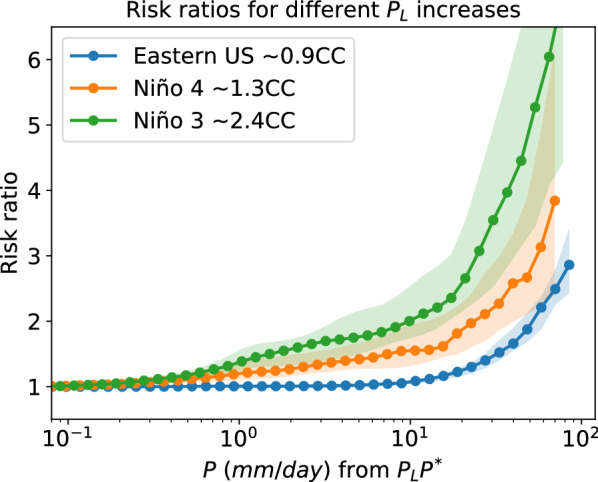
Risk ratio (2075–2099 vs. 1990–2014) in three regions with different $P_{L}$ increases: the Eastern United States (25$^\circ$N-48$^\circ$N,66$^\circ$W-103$^\circ$W), the Niño 4 region (5$^\circ$S-5$^\circ$N,160$^\circ$E-150$^\circ$W), and Niño 3 region (5$^\circ$S-5$^\circ$N,150$^\circ$W-90$^\circ$W). Solid lines show the multi-model mean and shading denotes the 25–75th percentiles spread across models. Risk ratios are calculated in scaled coordinates in each model prior to calculating the multi-model mean, and then scaled back by the multi-model mean increase in $P_{L}$. In the Eastern US, the multi-model mean $P_{L}$ increases from $P_{L}^{his}=15.1$mm in the historical case (1990–2014) to $P_{L}^{fut}=18.1$mm at the end of the century (2075–2099) for an increase of approximately $6\%$ per global mean temperature increase (multi-model mean of $3.96^\circ {\text{C}}$) (approx. $0.9\times CC$). Changes in $\tau _{P}$ are more modest, with multi-model mean of $\tau _{P}^{his}=0.64$ and $\tau _{P}^{fut}=0.68$. In the Niño 4 parameters in each period are $\tau _{P}^{his}=0.63$, $\tau _{P}^{fut}=0.6$, $P_{L}^{his}=14.8$mm, $P_{L}^{fut}=20.3$mm (increase of approx. $1.3\times CC$). In the Niño 3 region: $\tau _{P}^{his}=0.77$, $\tau _{P}^{fut}=0.75$, $P_{L}^{his}=12.7$mm, $P_{L}^{fut}=21.1$mm (increase of approx. $2.4\times CC$).

### Consequences of super-CC scaling for risk ratio

As seen in the previous section, global warming changes in daily precipitation intensity can be understood to first approximation by analyzing changes in the power law range exponent $\tau _{P}$ and precipitation scale $P_{L}$ across climates. Here we return to consequences for the changes in the frequency of extremes as measured by risk ratios.

Figure 6 shows the multi-model mean risk ratio in three regions that illustrate increases in extremes for different fractional increases in $P_{L}$ ($\gamma =\frac{\delta P_{L}}{P_{L}}$), with $\delta P_{L}=P_{L}^{fut}-P_{L}^{his}$ (i.e., $P_{L}^{fut}=(1+\gamma )P_{L}^{his}$). Here, the Eastern US experiences a multi-model increase of approximately 0.9*CC* ($6.2\%/$K), the Niño 4 region a multi-model increase of approximately 1.3*CC* ($9.4\%/$K), and the Niño 3 region a multi-model increase of approximately 2.4*CC* ($16.5\%/$K). In all cases, the risk ratio increases steeply for the largest events, with the biggest change in the region with the largest increment in $P_{L}$ (Niño 3 region). Under a SSP5 8.5 scenario, days with precipitation above 65mm increase by a factor larger than 6 (multi-model mean) in this region.

The shape of these risk ratios, as well as changes in frequency found in the observed record^7,24,26,34^ and global warming projections^11,14,25,27^, can be understood from changes in the daily precipitation probability distribution under the expected changes in its parameters. For the case of no change in the power law range exponent $\tau _{P}$, the risk ratio under the large $P_{r}$ limit can be written as (see Methods)

3 $$\begin{aligned} r(P_{r})\approx (1+\gamma )^{\tau _{P}}exp\left( \frac{\gamma }{(1+\gamma )}\frac{P_{r}}{P_{L}^{his}}\right) . \end{aligned}$$

That is, unlike increases in intensity, which have a linear dependence on $\gamma =\frac{\delta P_{L}}{P_{L}}$ (eq. 2), increases in frequency depend exponentially on a function that depends on increases in precipitation scale ($\frac{\gamma }{(1+\gamma )}\frac{P_{r}}{P_{L}^{his}}$) (Fig. S3a). This implies that even modest deviations above CC scaling can have large consequences for the frequency of the most extreme daily precipitation events. Figure S3b expands on the dependence of the risk ratio on $\gamma$ according to (3) under a typical configuration of probability distribution parameters. The range for which the multimodel risk-ratio evaluation is shown in Fig. 6 has been extended using the method of Fig. 2 (note that in scaled-coordinates the risk ratio is proportional to $exp(\frac{\gamma }{(1+\gamma )}P_{r}^{*})$), but is still limited by the requirement of a sufficient sample of extreme events in a 25 year period in historical climate in a given region. It is reasonable to expect the risk ratio for more extreme events to further increase with a leading dependence approximated by (3). Although models exhibit some departures from observed PDF shape in the low-medium event size range, the dependence illustrated in Fig. 6 typifies the medium-high event range behavior at a given multiple of CC scaling. The risk-ratio increases shown for the Niño-4 region exemplify frequency increases for regions exceeding 1.2CC (more than 40% of latitude bands in the deep tropics for ocean and land), while those shown for the Niño-3 region exemplify regions exceeding 2CC (more than 30% of tropical land.

## Discussion

In this study, we employ theory for precipitation probability distributions to improve estimates of changes in frequency of daily precipitation extremes under global warming in the CMIP6 ensemble and to interpret these. With the improved estimates, we find substantial regions of faster than CC scaling under warming, and identify consequences for the risk increase of the largest events.

There is a large model spread in the projected increases in frequency of daily precipitation intensity under global warming. In both observations and theory, leading behavior in the medium to high intensity range is governed by a dominant precipitation scale, controlled by the physics of the precipitating regime. While some CMIP6 models have errors in PDF shape that affect projections at low-moderate intensities, the shapes at medium-high intensity are more similar to each other and to observations in an analysis that controls for the precipitation scale^35^. Intermodel spread in this range can be reduced by rescaling the historical and global warming probability distributions by their historical precipitation scale. As an example, this simple technique can reduce by more than three times the projected model spread of the frequency of events larger than 45mm/day by the end of the century in the Amazon region. Furthermore, using a scaled coordinate extends the range of precipitation values for which changes in frequency can be projected in the multimodel ensemble. This provides a way to constrain projected increases in precipitation frequency for low-probability events.

As seen in previous studies^20,21^, the more extreme percentiles increase faster under global warming. In theoretical models^11,14,16,17^, the key scale $P_{L}$ controlling the large event tail scales with the size of moisture convergence fluctuations within precipitating events. Under a global warming scenario, changes in this single scale incorporate both thermodynamic and dynamic contributions to the precipitation probability distribution change: if there are no changes in the statistics of horizontal wind convergence (thermodynamic contribution only), this scale would increase approximately following a Clausius-Clapeyron scaling. When there are dynamical changes, i.e., in wind convergence associated with precipitation events, this scale will depart regionally from a CC scaling, with regions like the tropical Pacific or the Sahel, for example, having super CC increases. In most regions, the daily precipitation PDF power law range gets steeper with warming, affecting low-medium intensity events, in which cases changes in the precipitation scale $P_{L}$ provide an upper limit estimate for changes in high percentiles of intensity.

Fractional changes in extreme percentiles intensity under global warming $\frac{\delta P_{q}}{P_{q}}$ tend to follow (usually being slightly smaller) fractional changes in $P_{L}$ ($\gamma =\frac{\delta P_{L}}{P_{L}}$). Corresponding to this, changes in extreme precipitation frequency evaluated using risk ratios increase exponentially with $\gamma$. Even small differences in $P_{L}$ increases have exponentially large effects in the frequency of extreme events. These disproportionate increases in the risk ratio of rare events for even modestly super-CC scaling have implications for societal impacts. Projected changes in which some regions experience super-CC scaling, even if CC represents the median scaling, yield a larger increase in the probability of some region experiencing a previously rare event than if all regions had CC scaling. The results here suggest that occurrence of such regions of substantially super-CC scaling is likely, even if models do not necessarily agree on the precise location, especially for tropical land regions.

## Methods

### Model simulations and observational datasets

We use daily temperature and daily precipitation from the first available (r1i1p1f1, unless otherwise noted) historical^64^ and SSP5 8.5^65^ simulations from 32 models participating in the Coupled Model Intercomparison Project Phase 6 (CMIP6)^64^. The models used are: ACCESS-CM2, ACCESS-ESM1-5, BCC-CSM2-MR, CAMS-CSM1-0 (r2i1p1f1), CanESM5, CESM2, CESM2-WACCM, CMCC-CM2-SR5, CNRM-CM6-1 (r1i1p1f2), CNRM-CM6-1-HR (r1i1p1f2), CNRM-ESM2-1 (r1i1p1f2), EC-Earth3, EC-Earth3-Veg, GFDL-CM4, GFDL-ESM4, HadGEM3-GC31-LL (r1i1p1f3), HadGEM3-GC31-MM (r1i1p1f3), INM-CM4-8, INM-CM5-0, IPSL-CM6A-LR (r2i2p2f1), KACE-1-0-G, KIOST-ESM, MIROC6, MIROC-ES2L, MPI-ESM1-2-HR, MPI-ESM1-2-LR, MRI-ESM2-0, NESM3, NorESM2-LM, NorESM2-MM, TaiESM1, UKESM1-0-LL (r1i1p1f2). We perform all calculations in the original model grids, except for CNRM-CM6-1-HR, EC-Earth3, EC-Earth3-Veg, HadGEM3-GC31-MM, MPI-ESM1-2-HR, which are regridded to a 1$^\circ$-1$^\circ$ grid prior to analysis. Output from the native grids are regridded to a 1$^\circ$-1$^\circ$ grid to be displayed as maps (Figs. 1 and 5).

Regions used to illustrate the PDFs and their changes under warming are chosen to span geographical and climate diversity, including examples in the tropics, mid-latitudes, ocean and land regions, and in relatively dry and relatively wet zones. Two observational datasets (TRMM-3B42^40^ and PERSIANN^41^) are used to summarize the range of behaviors seen in detailed comparisons of different daily precipitation observational datasets^35,37^. The shape of daily precipitation probability distributions and the approximate collapse of the large event tail shown in Fig. 2c,f,i, are fairly insensitive to the dataset used^35^.

### Calculation of daily precipitation probability distributions parameters

Consider the case of a Gamma distribution, as used in previous studies^16,33^,

4 $$\begin{aligned} f(P; P_{L}, \tau _{P})=\frac{1}{\Gamma (1-\tau _{P})P_{L}^{1-\tau _{P}}}P^{-\tau _{P}}exp\left( -\frac{P}{P_{L}}\right) \end{aligned}$$

as an approximation for the daily precipitation probability distribution. Estimators for the parameters $\tau _{P}$ and $P_{L}$ using the method of moments are

5 $$\begin{aligned} P_{L}=\frac{\sigma _{P}^{2}}{{\bar{P}}_{wet}}; \,\,\,\,\, \tau _{P}=1-\frac{{\bar{P}}^{2}}{\sigma _{P}^{2}}, \end{aligned}$$

where ${\bar{P}}_{wet}$ and $\sigma _{P}^{2}$ are the daily precipitation mean and variance, respectively, over wet days (here taken as days with $P\ge 0.1mm$).

Several caveats must be noted on the Gamma distribution: for large enough *P* the extreme tail may deviate from exponential^66–69^ as seen in Fig. 2c,f,i. Analyses using extreme value theory for block maxima (e.g., annual maximum precipitation) typically point to parent distributions (i.e., the distribution of daily intensities examined here) with heavier extreme tails^70–72^. Note that a parent Gamma distribution is not necessarily inconsistent with a heavier than exponential distribution of block maxima if the slow convergence rate to the asymptotic extreme value distribution is taken into consideration^67^.

We underline that the Gamma distribution is used for reference, but one must consider robustness of properties of interest to departures from this. The key parameter for present purposes is $P_L$. When considering a distribution that departs from Gamma without an analytic form, as for observations or models, the usefulness of the scale estimator is determined by the extent to which collapse of PDFs to a common form in the medium-to large range occurs when rescaled by $P_L$. The scale given by the moment ratio (5a) is typically only modestly affected by small departures from Gamma at large intensities (e.g., Fig. 2), but can be affected by differing departures from Gamma in the low-intensity range. The moment estimator and a $P_L$ estimator from only the medium-large event range tend to yield similar behavior^46^.

### Bootstrap error bars on $P_L$, PDFs and super-CC fractions

For individual models shown in Fig. 2a,b, sampling variability is assessed by a one-year block bootstrap^35,73^, randomly picking (with replacement) 25 years of data from the underlying timeseries and recomputing the probability distributions from N = 100 realizations of the bootstrap timeseries. Shading denotes the 5th and 95th percentile for each PDF bin of these distributions. The 5th-95th percentile of $P_{L}$ estimations from these realizations are also shown. These uncertainty ranges do depend on the geographical size and climatology of the region examined. Increased geographic size tends to reduce uncertainty range analogous to increasing the size of the timeseries; spatial correlation of precipitation is important factor in this (uncertainty does not increase as rapidly as if spatial points were independent). The spatial relations (as well as sub-yearly temporal correlations) are preserved in the bootstrap estimate. Regions with low precipitation tend to have higher uncertainty in $P_{L}$ due to fewer events. The Nino-3.4 region represents a region of relatively high estimation uncertainty and yet the ranges in Fig. 2a,b illustrate estimation uncertainty is modest compared to the intermodel uncertainty in $P_L$.

Bootstrap error bars for the multi-model mean fractional change in the precipitation scale in Fig. 5 are calculated by randomly picking (with replacement) one hundred realizations of the 32 models considered and recomputing estimations of the multi-model mean.

### Changes in percentiles

The analytical approximation for changes in percentiles for the case of the Gamma distribution (4) is derived as follows. The Gamma cumulative distribution $F(P; P_{L}, \tau _{P})$ is given by

6 $$\begin{aligned} F(P;P_{L},\tau _{P})=1-\Gamma \left( 1-\tau _{P},\frac{P}{P_{L}}\right) , \end{aligned}$$

where $\Gamma (y,z)=\frac{1}{\Gamma (y,0)}\int _{z}^{\infty }x^{y-1}exp(-x)dx$ is the incomplete Gamma function. From this, we can derive analytically expressions for wet-day and all-day percentiles and their change as a function of the parameters of the distribution. In what follows we make a distinction between percentiles and left-censored percentiles (denoted with the superscript $^{l}$). We use left-censored percentiles in Fig. 4 to avoid issues associated with the drizzle problem^48^.

For a given value of $P_{L}$ and $\tau _{P}$, the q-th wet-day percentile $P_{q}^{wet}$(with no left-censoring) value, such that $100F(P_{q}^{wet};P_{L},\tau _{P})=q$, is given by

7 $$\begin{aligned} P_{q}^{wet}=P_{L}\Gamma ^{-1}\left( 1-\tau _{P},1-\frac{q}{100}\right) . \end{aligned}$$

An all-day percentile $P_{q}^{all}$ can be written as

8 $$\begin{aligned} P_{q}^{all}=0&,\,\,\,\text {for}\,\,\,q<100f_{dry}\nonumber \\ P_{q}^{all}=P_{\frac{(q-100f_{dry})}{f_{wet}}}^{wet}&,\,\,\,\text {for}\,\,\,q\ge 100f_{dry}, \end{aligned}$$

where $f_{wet}$ and $f_{dry}$ are the fraction of wet and dry days (prior to left-censoring) and $f_{dry}=1-f_{wet}$.

Evaluating (7) under historical ($P_{L}$, $\tau _{P}$) and global warming ($P_{L}+\delta P_{L}$, $\tau _{P}+\delta \tau _{P}$) conditions, we find that the fractional change in wet-day percentiles is approximately given by

9 $$\begin{aligned} \frac{\delta P_{q}^{wet}}{P_{q}^{wet}}=\frac{\delta P_{L}}{P_{L}}+\left[ \frac{\Gamma ^{-1}(1-(\tau _{P}+\delta \tau _{P}),1-\frac{q}{100})}{\Gamma ^{-1}(1-\tau _{P},1-\frac{q}{100})}-1\right] , \end{aligned}$$

which has the form given by equation 2, with function *G* given by the second term in the right-hand-side of the previous equation. A projected steepening of the power law range (i.e., increase in $\tau _{P}$) under global warming in most regions (Fig. S2a) implies that *G* is negative at every percentile level, although with a diminishing effect for the most extreme ones. Under these conditions, this implies that $\frac{\delta P_{L}}{P_{L}}$ is an upper limit for fractional increases in $P_{q}^{wet}$. In principle, a similar expression for the fractional change in all-day percentiles could be derived from (8), but with the added complication that it also depends on changes in wet-day fraction.

A left-censored wet-percentile $P_{q_{left}}^{l,wet}$ is related to a wet-percentile $P_{q}^{wet}$ as follows

10 $$\begin{aligned} P_{q_{left}}^{l,wet}=P_{\frac{100(q-q*)}{100-q*}}^{wet}, \end{aligned}$$

where *q* is the percentile considering all non-zero values (including below the wet-day threshold), and $q^{*}$ is the percentile that corresponds to the wet-day threshold given by

11 $$\begin{aligned} q^{*}=\Gamma \left( 1-\tau _{P};\frac{P_{thr}}{P_{L}}\right) , \end{aligned}$$

where $P_{thr}$ is the threshold chosen to define a wet day (0.1mm/day in this study).

Finally, a left-censored all-day percentile is given by

12 $$\begin{aligned} P_{q}^{l,all}=0,\,\,\,&\text {for}\,\,\,q<100f_{l,dry},\nonumber \\ P_{q}^{l,all}=P_{\frac{(q+q*-f_{l,dry})}{q*+f_{l,wet}}}^{wet},\,\,\,&\text {for}\,\,\,q\ge 100f_{l,dry}, \end{aligned}$$

where $f_{l,dry}$ and $f_{l,wet}$ are now the fraction of dry and wet days, respectively, when left-censoring is taken into account —i.e., days with $P<P_{thr}$ are considered dry in this measure. In Fig. 4 we plot the left-censored changes in wet-day percentiles (Fig. 4a) and all-day percentiles (Fig. 4b). To do this, we calculate expressions (10) and (12) under historical and global warming conditions, and then calculate the fractional change.

### Analytical calculation of risk ratios

A risk ratio *r* conditioned on wet-day occurrence is defined as the ratio between global warming (SSP5 8.5 scenario here) and historical daily precipitation probability exceedances ($1-F$, with *F* the cumulative density function) in a given location^7,34^. For the analytical case presented in (3) these are calculated as

13 $$\begin{aligned} r(P)=\frac{\Gamma \left( 1-\tau _{P}^{fut};\frac{P}{P_{L}^{fut}}\right) }{\Gamma \left( 1-\tau _{P}^{his};\frac{P}{P_{L}^{his}}\right) }, \end{aligned}$$

where the superscript $^{his}$ and $^{fut}$ denote parameters calculated in the historical and SSP5 8.5 simulations respectively. For large $\frac{P}{P_{L}}$, we can approximate^74^

14 $$\begin{aligned} \Gamma \left( 1-\tau _{P};\frac{P}{P_{L}}\right) \approx \left( \frac{P}{P_{L}}\right) ^{-\tau _{P}}exp\left( -\frac{P}{P_{L}}\right) \left[ 1+O\left( -\frac{\tau _{P}}{\left( \frac{P}{P_{L}}\right) }\right) +...\right] . \end{aligned}$$

Keeping only the first term, and defining $\gamma =\frac{\delta P_{L}}{P_{L}^{his}}$, with $P_{L}^{fut}=P_{L}^{his}(1+\gamma )$, we get in the general case

15 $$\begin{aligned} r_{P}\approx \frac{P^{\tau _{P}^{his}}}{P^{\tau _{P}^{fut}}}\frac{(P_{L}^{fut})^{\tau _{P}^{fut}}}{(P_{L}^{his})^{\tau _{P}^{his}}}exp\left( \frac{\gamma P}{P_{L}^{fut}}\right) . \end{aligned}$$

In the simpler case in which $\tau _{P}^{his}=\tau _{P}^{fut}=\tau _{P}$, we get

16 $$\begin{aligned} r_{P}\approx (1+\gamma )^{\tau _{P}}exp\left( \frac{\gamma P}{P_{L}^{fut}}\right) , \end{aligned}$$

which is the approximation used in Fig. S3a for the analytical case.

## Supplementary Information

Supplementary Information.

## Data Availability

CMIP6 simulations can be accessed at https://esgf-node.llnl.gov/search/cmip6/. TRMM-3B42 and PERSIANN precipitation datasets can be accessed at https://data.ipsl.fr/catalog/srv/eng/catalog.search#/metadata/6e546110-2bd8-4f42-b0b9-8a65b49274f6^77^.
