# Supplementary material for: Regionally high risk increase for precipitation extreme events under global warming
Source: Sci Rep. 2023 Apr 5;13:5579. doi: 10.1038/s41598-023-32372-3 (PMC10076280; doi:10.1038/s41598-023-32372-3)
Supplement: Supplementary file 1 — Supplementary Information. [file 41598_2023_32372_MOESM1_ESM.pdf]

Supplementary Figures for

Regionally high risk increase for precipitation extreme events under global warming

Cristian Martinez-Villalobos and J. David Neelin

March 13, 2023

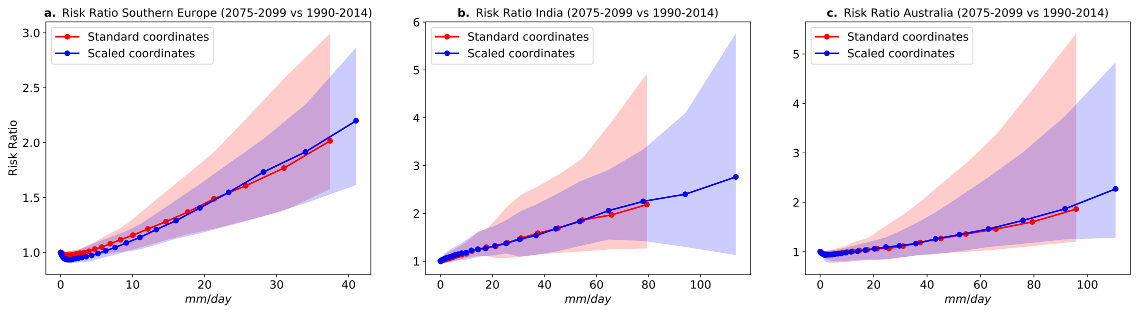

Figure S1: Same as Fig. 3c,d in the main text, but for **a.** Southern Europe (40N-50N,0E-20E), **b.** India (5N-25N,65E-90E), and **c.** Australia (10S-40S,110E-155E)

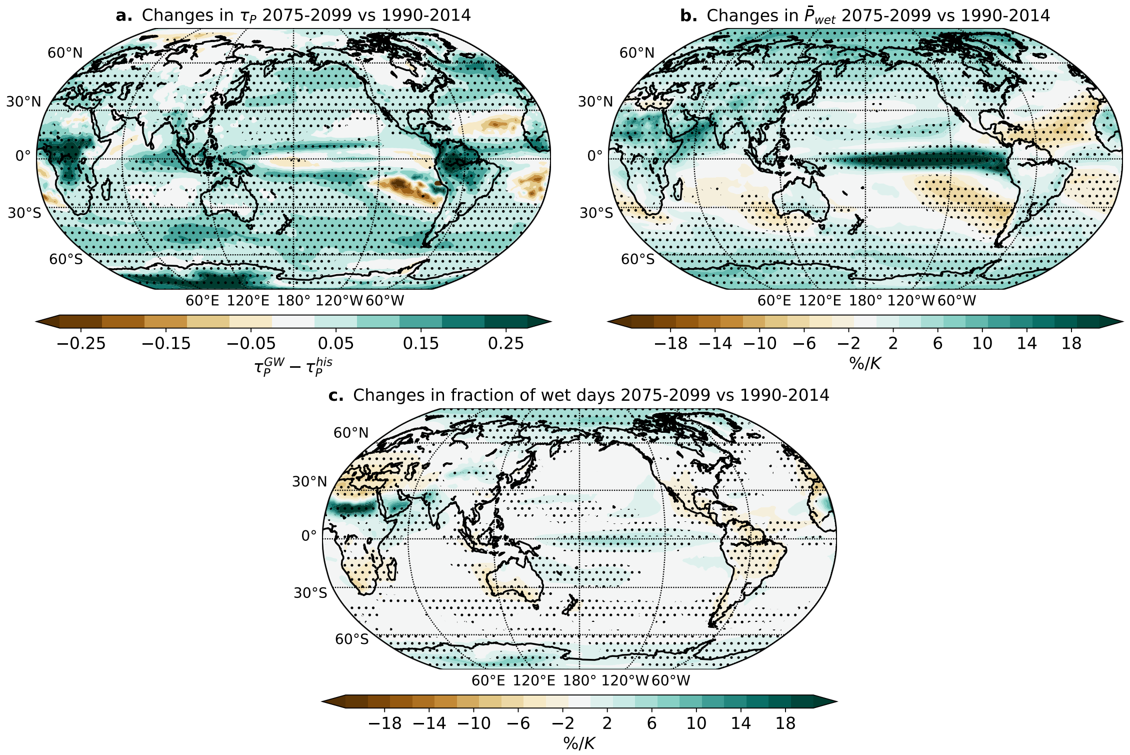

Figure S2: **a.** Multi-model mean of the absolute change in power law range exponent τ_P comparing 2075-2099 (τ_P^{GW}) and 1990-2014 (τ_P^{his}). **b.** (**c.**) Multi-model mean fractional change of the mean over wet days $\bar{P}_{wet}$ (fraction of wet days) comparing 2075-2099 vs 1990-2014. In **b** and **c** changes are normalized by each model global mean temperature change prior to aggregating. Dots show regions in which at least 80% of the models agree in the sign of the change.

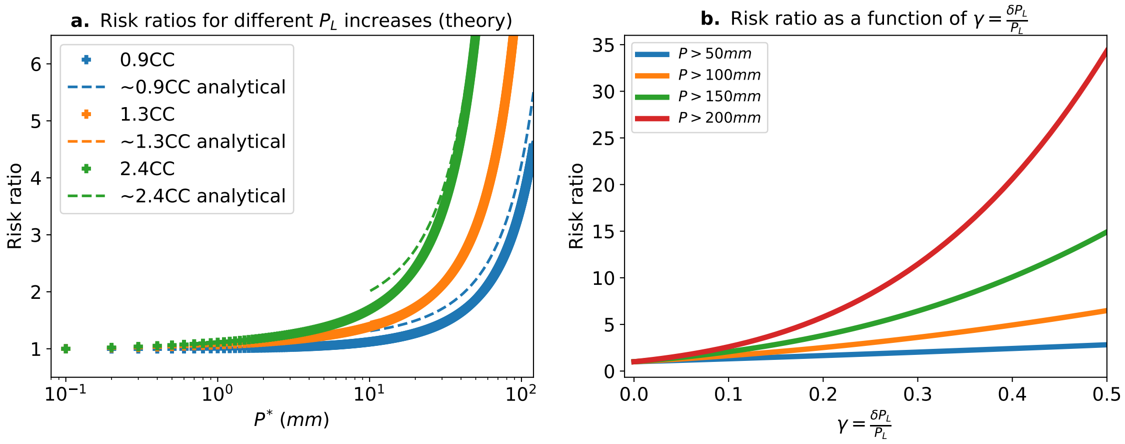

Figure S3: **a.** Analytical risk ratios according to equation 13 in main text (crosses), and approximation for large $\frac{P}{P_L}$ (assuming no change in τ_P) (equation 3 in main text) corresponding to the same changes in parameters as in Figure 6 in main text (see caption). **b.** Risk ratio for different values of P_r as a function of $\gamma = \frac{\delta P_L}{P_L}$ according to approximation given in equation 3 in main text. In this example $\tau_P^{his} = \tau_P^{fut} = \tau_P = 0.5$, $P_L^{his} = P_L = 20mm$, and $P_L^{fut} = (1 + \gamma)P_L$.
